# Seaweed sulfated polysaccharides regulate the gut microbiota-mucosal barrier-innate immunity axis: structural heterogeneity, immunological evidence, and translational prospects

**DOI:** 10.3389/fimmu.2026.1920049

**Published:** 2026-09-10

**Authors:** Wei Liu, Meijing Zhang, Jianan Huang, Sihan Liu, Ran Chen, Yingqiang Li, Jinlin Liu, Rui Jia

**Affiliations:** 1 School of Environmental and Chemical Engineering, Shanghai University, Shanghai, China; 2 College of Oceanography, Fujian Polytechnic Normal University, Fuzhou, China; 3 School of Basic Medical Sciences, Naval Medical University, Shanghai, China; 4 Southern Zhejiang Key Laboratory of Crop Breeding, Wenzhou Academy of Agricultural Sciences, Wenzhou, China; 5 Project Management Office of China National Scientific Seafloor Observatory, Tongji University, Shanghai, China; 6 Key Laboratory of Marine Ecological Monitoring and Restoration Technologies, East China Sea Ecological Center, Ministry of Natural Resources, Shanghai, China; 7 College of Oceanography and Ecological Science, Shanghai Ocean University, Shanghai, China

**Keywords:** seaweed sulfated polysaccharides, gut microbiota, mucosal barrier, innate immunity, porphyran, ulvan

## Abstract

Seaweed sulfated polysaccharides, fucoidan, carrageenan, ulvan, and porphyran, are structurally diverse marine glycans that modulate the gut microbiota, mucosal barrier, and innate immunity, yet the literature remains fragmented across classes, models, and processing histories. This review synthesizes evidence on their structure-function relationships, focusing on microbial enzymatic accessibility, selective fermentation, metabolite remodeling, barrier regulation, and immune signaling, while also assessing translational barriers. A systematic literature search prioritized mechanistic studies, structural analyses, animal models, human trials, and high-quality reviews. Our analysis reveals that porphyran provides the clearest microbiota–barrier coupling, fucoidan and carrageenan give the richest, but most heterogeneous, immune readouts, and ulvan is an emerging class with growing intestinal evidence. Molecular weight, sulfation pattern, and degradation history consistently shape microbial utilization and downstream effects across all classes. Disease contexts with the strongest support include inflammatory bowel disease, constipation, barrier injury, and infection-driven intestinal inflammation. Time-dependent immune shifts have been reported, but durable epigenetic memory remains unproven, and extra-intestinal evidence through gut-liver and gut-brain axes is still preliminary. We conclude that these polysaccharides are best understood as structurally defined substrates that bridge the microbiota, barrier, and immunity; future progress toward clinical translation will depend on stricter standardization, integrated mechanistic designs, and robust human data.

## 1 Introduction

Seaweed polysaccharides are among the most mature and versatile molecular families in marine natural product research. Early work focused mainly on their gelling, adhesive, chelating, and rheological properties, but advances in structural analysis, enzymology, and omics technologies have shifted the field from “extractable marine colloids” toward “definable bioactive macromolecules.” In this process, brown-seaweed fucoidan, red-seaweed carrageenan and porphyran, and green-seaweed ulvan, together with closely related alginate and laminarin, have increasingly been shown to display anticoagulant, antiviral, antioxidant, immunomodulatory, and tissue-protective activities (1–5).

Unlike conventional dietary fiber, the biological effects of these molecules are strongly structure dependent. Differences in species origin, processing, and purification route change molecular weight, sulfation pattern, and coexisting components, which means that materials bearing the same name do not always behave identically across studies (1–3). The key question is therefore not simply whether a seaweed polysaccharide is active, but what structure is present, in which system, and through which route the observed effect arises.

This structural diversity results in a wide range of immune outcomes that are often difficult to compare directly. Trained immunity provides a useful framework for interpreting some of these observations. The concept, originally introduced by Netea and colleagues, emphasizes that innate immune cells can undergo metabolic and epigenetic reprogramming after an initial stimulus and respond more rapidly or more strongly when challenged again (6, 7). For seaweed sulfated polysaccharides, however, the evidence is not yet strong enough to claim that they are bona fide trained-immunity inducers. At present, they are better viewed as molecules that can reshape the microbiota, metabolites, and mucosal barrier, thereby altering the microenvironment in which innate immune cells operate.

Host immune function is closely intertwined with the gut microbiota. Rooks and Garrett highlighted microbial metabolites as key mediators linking diet and host immunity, while Hehemann et al. demonstrated that red seaweed glycans can be directly catabolized by gut bacteria, thereby establishing a molecular connection between seaweed polysaccharides and microbial metabolism (8–10). Consequently, seaweed sulfated polysaccharides are not merely marine natural products; they represent a class of structured substrates that can be selectively utilized by the gut microbiota and, in turn, influence mucosal immunity and tissue homeostasis, suggesting that these polysaccharides may modulate host immune function through microbiota-mediated mechanisms.

The present review is organized around four interconnected levels: the major polysaccharide classes and their structural heterogeneity; their interactions with the gut microbiota and microbial enzyme systems; the innate immune and mucosal barrier pathways involved; and the disease-relevant evidence together with the key translational bottlenecks that remain to be addressed.

The literature covered in this Review was identified through searches of the Web of Science core collection using keywords including fucoidan, carrageenan, ulvan, porphyran, alginate, laminarin, gut microbiota, mucosal barrier, innate immunity, trained immunity, colitis, constipation, sulfatase, and porphyranase. We prioritized English-language articles and included landmark mechanistic studies, structural analyses, animal experiments, a limited number of human trials, and high-quality reviews. The evidence was organized along a unified analytical chain, from structural features to microbial metabolism, barrier outcomes, and immune readouts, so that studies differing in source material, processing, and model systems could be compared and key knowledge gaps identified.

## 2 Major types and structural heterogeneity of seaweed sulfated polysaccharides

### 2.1 Fucoidan: a structurally diverse family under one name

Fucoidan is the most extensively studied of the seaweed sulfated polysaccharides, but the term does not denote a single, uniform molecule. Fucoidan preparations from different brown algae, harvest seasons, and extraction or purification workflows can vary substantially in monosaccharide composition, glycosidic linkages, branching density, molecular weight, sulfation position, acetylation level, and the presence of uronic acid or galactose residues (11–15). This structural heterogeneity is a primary source of the functional variability observed across studies. The sulfated fucans that constitute the backbone typically confer a net negative charge to the molecule, which is thought to influence its interactions with both microbial enzymes and host cell surface receptors (1, 16, 17). Depending on the algal species, fucoidan consists predominantly of α-L-fucose repeating units, with sulfation occurring most frequently at the C-2, C-3, and C-4 positions, albeit in varying substitution patterns (14). The core linkage also differs by taxonomic group: fucoidans from Chordariales and Laminariales species are primarily composed of (1 → 3)-linked α-L-fucopyranose units, whereas those from Ascophyllum and Fucus exhibit an alternating (1 → 3) and (1 → 4) linkage pattern (18, 19). Total sulfur content or average molecular weight alone is therefore insufficient to capture the true chemical identity of the material; instead, a combination of Nuclear Magnetic Resonance (NMR), chromatographic fractionation, and enzymatic cleavage provides a more reliable analytical framework (1–3, 20).

This heterogeneity directly affects how the biological literature should be interpreted. Some fucoidan samples approximate regular linear sulfated fucans, whereas others are highly branched composite structures containing uronic acids and multiple additional substituents. Even when preparations share a common extraction label, chemically distinct materials may not be functionally equivalent. The central task in fucoidan research, therefore, is not to compare samples in a binary “active versus inactive” manner, but to determine which structural motifs are preserved in a given source, fraction, or degradation stage, and to establish how those motifs participate in specific receptor recognition events and downstream signaling cascades (2, 3, 20). Without such structural clarity, cross-study comparisons remain inherently limited, and the mechanistic interpretation of fucoidan bioactivities risks being reduced to phenomenological descriptions rather than structure-based principles.

### 2.2 Carrageenan: subtype, modification, and functional boundaries

Carrageenan, derived from red algae, is a collective term for a family of high-molecular-weight, hydrophilic sulfated galactans composed of alternating D-galactose and 3,6-anhydro-galactose (3,6-AG) units linked by alternating α-1,3 and β-1,4 glycosidic bonds, with ester-sulfate content ranging from 15% to 40% that confers a net anionic character (21, 22). Based on sulfation degree, source species, and solubility, carrageenans can be classified into six basic forms, namely kappa (κ-), iota (ι-), lambda (λ-), mu (μ-), nu (ν-), and theta (θ-) carrageenan, among which κ-, ι-, and λ-carrageenan are the most commercially and pharmaceutically relevant (23, 24). These three major subtypes differ by the number of ester-sulfate groups per repeating disaccharide unit: one for κ-, two for ι-, and three for λ-carrageenan. However, the chemical heterogeneity of carrageenan is not solely a function of subtype; it is also influenced by algal source, tissue age, harvest season, and extraction conditions (5, 25). Even within a single species such as *Kappaphycus alvarezii*, the most important commercial source of κ-carrageenan, chemical composition can vary among strains. Across six strains, total carbohydrates ranged from 51.6% to 55.8% on a dry basis, while ash, sulfate groups, proteins, insoluble aromatics, galacturonic acid, hydroxymethylfurfural, and lipids ranged from 14.6–17.2%, 9.6–10.8%, 2.3–3.8%, 1.5–3.4%, 0.9–1.5%, 2.8–4.5%, and 0.2–1.3%, respectively (the unaccounted residual may include cell-wall components not fully recovered, additional neutral sugars or uronic acids not quantified, soluble low-molecular-weight compounds, and procedural losses inherent to each method) (21, 22).

This structural and compositional variability has profound implications for how carrageenan is interpreted in food versus biomedical research, a distinction that is often overlooked. In food applications, the high-molecular-weight native forms are valued for their gelling, thickening, and stabilizing properties, and their safety profile is well established. In cell-based and animal studies, however, lower-molecular-weight fractions or chemically modified derivatives have been reported to exhibit antiviral, immunostimulatory, or inflammatory-modulating activities (26–28). These two bodies of evidence are not necessarily contradictory; they reflect the fundamental principle that the biological behavior of carrageenan is not an intrinsic property of the “carrageenan” label, but is contingent upon molecular weight, sulfation pattern, and degradation history. The most productive way to discuss carrageenan, therefore, is to recognize that its functional range spans a continuum determined by structural parameters, and that meaningful cross-study comparison requires explicit reporting of subtype, molecular weight, sulfation density, and processing history, a standard that remains inconsistently met in the current literature.

### 2.3 Ulvan: a heterogeneous backbone strongly shaped by extraction conditions

Ulvan, the representative sulfated heteropolysaccharide of green algae, is derived from the cell walls of *Ulva* species, including taxa formerly placed in *Enteromorpha* (e.g., *U. lactuca*, *U. rigida*, *U. armoricana*, and *E. linza*). A variety of extraction methods have been developed for ulvan, including hot water extraction, hot water with calcium chelating agents (e.g., sodium oxalate or acidified ammonium oxalate), hot water with ethanol wash, enzyme-assisted extraction, and acidic extraction, each of which can influence the molecular weight, sulfation pattern, and impurity profile of the final product. Chemically, ulvan is a water-soluble sulfated heteropolysaccharide built predominantly from repeating ulvanobiuronic acid 3-sulfate motifs (A3S and B3S), together with xylose-containing variants such as U3S and U2′S,3S. In contrast to carrageenan, ulvan does not map neatly onto a carrageenan-like subtype classification system; rather, differences in source, cultivation conditions, and extraction procedure mainly alter monosaccharide composition, sulfate distribution, and molecular weight. This structural heterogeneity likely contributes to the considerable variability reported for its immunomodulatory, antioxidant, and intestinal-protective activities (3, 29–31).

Beyond its direct bioactivities, ulvan has also attracted interest for its material properties. It can form gels, crosslink, and be formulated into thermosensitive or injectable hydrogels, making it a promising candidate for intestinal protection, local delivery, and wound-related applications (3, 31). At the mechanistic level, however, the microbial degradation systems for ulvan, including the polysaccharide utilization loci (PULs) and sulfatases involved, remain poorly understood compared with the relatively well-characterized enzymatic pathways for porphyran and carrageenan, and the relationship between ulvan’s chemical heterogeneity and its selective fermentation by gut microbiota is only beginning to be explored (30, 32). Future work will need to clarify which structural features of ulvan are responsible for its immunomodulatory effects and how its material properties influence its behavior in the intestinal tract.

### 2.4 Porphyran and agar-related galactans: the clearest case of microbiota interaction

Porphyran, mainly obtained from Porphyra/Pyropia species (formerly Porphyra), is a sulfated galactan. Compared with agar, porphyran retains sulfate substitutions, which make it accessible to porphyranases and related microbial enzyme systems. Pioneering studies by Hehemann et al. established porphyran as one of the clearest examples of a seaweed polysaccharide that directly connects diet, microbial catabolism, and host physiology (2, 9, 10).

Functionally, porphyran is particularly informative because it links structural variation, microbial degradation, and mucosal responses. Partially hydrolyzed porphyran can retain key backbone fragments and still modulate the microbiota and improve barrier function in colitis models, suggesting that moderate depolymerization does not necessarily abolish activity and may enhance colonic accessibility and microbial utilization (33).

### 2.5 Structural parameters as the key to interpretation

**Figure 1:**
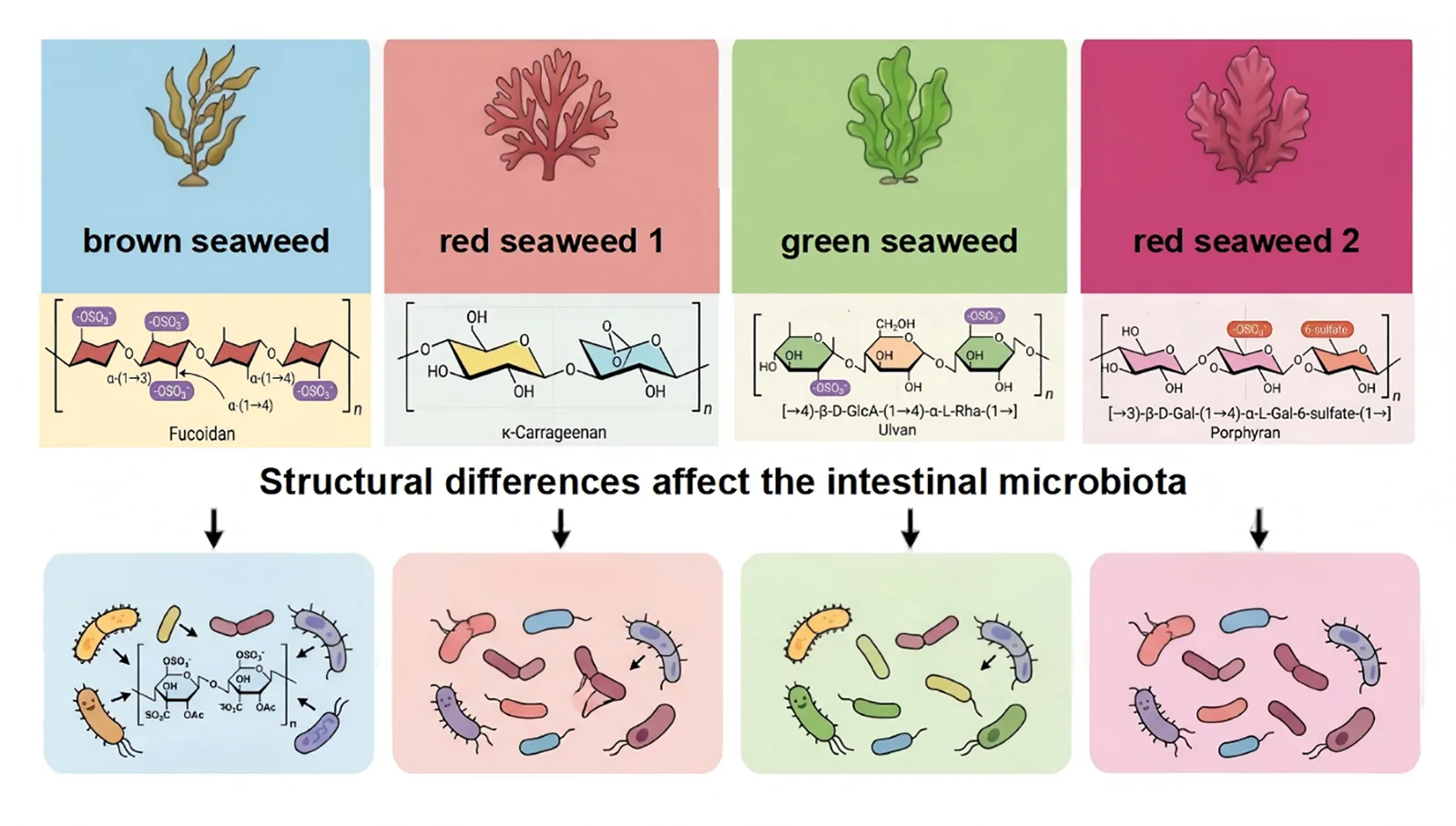
The structural differences of four main algal polysaccharides affect the intestinal microbiota.

If seaweed sulfated polysaccharides are classified only by name, much of the biological variability is obscured (**Figure 1**). A more useful approach is to consider molecular-weight distribution, sulfation degree, sulfate position, backbone linkage pattern, side chains and substituents, deacetylation or oxidation state, together with extraction and fractionation procedures. Sonication, enzymatic digestion, microwave treatment, acid or alkali treatment, and oligosaccharide preparation can all alter the structural phenotype of the same nominal molecule, and thereby change fermentation behavior, receptor recognition, and tissue-level effects (3, 34–39). In the sections that follow, the evidence is therefore discussed in terms of source, structure, processing, and model rather than by name alone.

## 3 Interactions between seaweed sulfated polysaccharides and the gut microbiota

### 3.1 Selective fermentation is the starting point of the host immune axis

After seaweed polysaccharides enter the digestive tract, the first changes usually occur not in host immunity but in substrate accessibility and microbial ecology. Existing reviews show that the fermentation of alginate and laminarin is relatively stable and is often accompanied by increased short-chain fatty acids and lower levels of some putrefactive metabolites, whereas the outcomes for fucoidan, carrageenan, and ulvan depend much more strongly on source, molecular weight, and sulfation pattern and therefore show greater structural selectivity (40, 41). From this perspective, seaweed polysaccharides are better understood as substrates recognized by specific microbes than as ordinary dietary fibers that anyone can use uniformly.

Microbial metabolism matters for much more than a simple change in taxa. Short-chain fatty acids, bile acids, ammonia, phenolic metabolites, indoles, and H_2_S all influence epithelial energy supply, mucus turnover, tight-junction stability, and inflammatory thresholds (8, 40). When evaluating whether a seaweed polysaccharide has a genuine intestinal-health effect, metabolite output and tissue endpoints are therefore more informative than the abundance of any single bacterial genus.

Baseline microbiota composition is another factor that may modify the response to seaweed sulfated polysaccharides. Human gut communities described as enterotype-like states differ particularly in the relative abundance of *Bacteroides* and *Prevotella* and are associated with long-term dietary patterns (42, 43). These differences may influence the extent to which a complex seaweed polysaccharide can be degraded, because its utilization requires substrate-matched polysaccharide utilization loci and carbohydrate-active enzymes, including glycoside hydrolases, carbohydrate esterases, and, for sulfated glycans, sulfatases (44, 45). The best-characterized human-gut examples are strain-and substrate-specific. *Bacteroides plebeius* carries a porphyran utilization locus containing GH16 and GH86 family β-porphyranases; *Bacteroides uniformis* NP1 possesses an agarose-utilization pathway (46); and *Bacteroides thetaiotaomicron* VPI-3731 has been shown to grow on carrageenan in substrate-screening experiments. Communities enriched in *Bacteroides* may therefore have greater potential to initiate the degradation of some marine glycans, whereas communities with limited primary-degradation capacity may depend more on secondary utilization after oligosaccharides have been released. The same polysaccharide preparation may consequently produce different fermentation profiles and downstream barrier or immune responses in hosts with different baseline microbiota. Future studies on the immunomodulatory effects of algal sulfated polysaccharides should routinely report baseline gut microbiota composition. This practice will help clarify how these polysaccharides modulate immune outcomes through the intestinal mucosal barrier.

### 3.2 Structural determinants and microbial enzyme systems governing the utilization of seaweed sulfated polysaccharides

The utilization of algal sulfated polysaccharides depends on the compatibility between their backbone structure and the catalytic specificity of microbial carbohydrate-active enzymes. This process generally requires coordinated activities of glycoside hydrolases or polysaccharide lyases, sulfatases, carbohydrate esterases, transport proteins, and regulatory components encoded within polysaccharide utilization loci (PULs). Therefore, the presence of a PUL or a taxonomic group alone does not prove that a microorganism can utilize an intact algal polysaccharide; substrate-specific growth, gene expression, enzymatic activity, or metabolite production should also be demonstrated (2, 9, 10). Whether a seaweed glycan can enter the host-microbe interaction chain therefore depends first on whether the microbiota has the required CAZymes and sulfatases.

For fucoidan, enzyme recognition is strongly influenced by the arrangement of α-L-fucose residues, the α-(1→3) or α-(1→4) glycosidic linkages, the positions of sulfate ester groups, and the presence of branches or acetyl substitutions. Endo-fucanases from the GH107 and GH168 families have been shown to act on selected fucose-rich backbones, whereas GH29, GH95, and GH141 fucosidases remove terminal fucose residues during later degradation steps. Sulfatases, including members of the S1_17, S1_22, and S1_25 families, are required to remove specific sulfate ester groups and thereby improve access to the glycosidic backbone. For example, GH168 enzymes can recognize selected α-(1→3)-linked fucose structures containing 2-O-sulfate groups. These enzyme cascades have been characterized mainly in marine Verrucomicrobia and Planctomycetota (47, 48).

Carrageenan degradation is determined by the carrageenan subtype and by the position of sulfate substitution. κ-Carrageenan contains β-(1→4) and α-(1→3) linkages and is mainly characterized by 4-O-sulfated galactose and 3,6-anhydrogalactose residues, whereas ι-carrageenan contains additional sulfate substitution on the anhydrogalactose residue. GH16 κ-carrageenases and GH82 ι-carrageenases hydrolyze subtype-specific β-(1→4) linkages. The resulting oligosaccharides are further processed by sulfatases and α-(1→3)-3,6-anhydro-D-galactosidases from the GH127 and GH129 families. These enzyme systems were first resolved in marine bacteria. More recent work in ruminant intestinal microbiomes identified carrageenan-active PULs and GH16 subfamily 17 carrageenases, with Bacteroides-enriched fecal communities showing greater capacity for carrageenan utilization (23, 49–51).

Ulvan contains sulfated rhamnose, glucuronic acid, iduronic acid, and, in some preparations, xylose-containing residues. Its repeating units include Rha3S-GlcA and Rha3S-IdoA motifs connected mainly through β-(1→4) linkages. PL24, PL25, PL28, PL37, and PL40 ulvan lyases initiate degradation through β-elimination at linkages adjacent to uronic-acid residues, generating unsaturated uronic-acid termini. These products are subsequently processed by auxiliary hydrolases, including GH105 enzymes, while sulfatases remove sulfate groups from sulfated rhamnose or other substituted residues. The best-characterized ulvan-degrading systems have been identified in marine flavobacteria and related algal-associated bacteria, such as *Formosa agariphila*. Direct evidence for an equivalent ulvan-degrading system in mammalian gut bacteria remains limited (52).

Porphyran is composed mainly of alternating 3-linked β-D-galactose and 4-linked α-L-galactose-6-sulfate residues, with variable amounts of 3,6-anhydro-L-galactose and O6-methylated galactose. GH16 and GH86 β-porphyranases recognize and cleave the β-(1→4) linkage between β-D-galactose and α-L-galactose-6-sulfate, producing characteristic sulfated oligosaccharides (53). The released oligosaccharides are then further processed by sulfatases and other PUL-associated enzymes. Among the four polysaccharide classes discussed here, porphyran currently has the clearest direct evidence in the human gut: *Bacteroides plebeius* carries a porphyran-specific PUL containing GH16- and GH86-family porphyranases and can grow on porphyran. Subsequent comparative genomic work further showed that seaweed-glycan utilization genes have entered the human gut microbiome through multiple transfer events, although their distribution remains uneven across microbial lineages and host populations (9, 10, 54).

Taken together, the microbial groups best adapted to algal-polysaccharide utilization should be defined by their functional enzyme repertoire rather than by phylum or genus abundance alone. Bacteroides species carrying porphyran-specific PULs provide the strongest human-gut example, while Bacteroidota carrying CarPULs provide emerging evidence for carrageenan utilization in ruminants. Other gut bacteria may participate through cross-feeding after primary degraders release oligosaccharides.

### 3.3 Structure-function relationships in the microbial utilization of algal sulfated polysaccharides

The presence of appropriate enzymatic machinery does not, by itself, guarantee a biological effect; the accessibility of the polysaccharide to the colonic microbiota is equally critical. In model systems, the effects of seaweed polysaccharides on the microbiota are closely linked to their accessibility in the colon. Partially hydrolyzed porphyran has been shown in DSS-induced colitis models to increase Bacteroides, Muribaculum, and Lactobacillus, together with higher short-chain fatty acid levels, a thicker mucus layer, and restored tight-junction proteins, suggesting a dual action through both substrate utilization and barrier protection (33). A similar selective-utilization pattern reported for a sulfated galactan from Gracilaria fisheri also supports the idea that moderate processing may make these molecules more suitable for colonic use (55). By contrast, non-sulfated or weakly sulfated molecules such as alginate and laminarin are more likely to form stable fermentation substrates in the gut and therefore display more regular effects on the microbiota and metabolite profile (40, 56). Sulfated polysaccharides are not less valuable; their function simply depends more heavily on fine structural definition and the relevant enzyme repertoire, which makes them especially suitable for well-defined materials and local action sites.

The metabolite profiles generated by different algal sulfated polysaccharides differ substantially. Porphyran and moderately degraded porphyran tend to be associated with increased short-chain fatty acid production, together with restoration of mucus thickness and tight-junction proteins in colitis models (33). Fucoidan-related outcomes are more variable. Its effects on short-chain fatty acids (SCFAs) remodeling and bile-acid-related signaling have been reported, but both appear to depend on molecular weight and sulfation pattern, with source material adding further variation (41, 57). Ulvan, because of its rhamnose- and uronic-acid-rich structure, may favor distinct fermentation routes and has been linked to amino-acid-related metabolites such as arginine and S-adenosylmethionine in constipation models (58). Carrageenan requires the most cautious interpretation because native high-molecular-weight food-grade forms, degraded low-molecular-weight fractions, and subtype-specific oligosaccharides may produce different inflammatory and metabolic outcomes. Thus, metabolite remodeling should be analyzed by polysaccharide class, degradation state, and host microbial background rather than treated as a generic increase in bioactive metabolites (59, 60).

### 3.4 The endpoint of microbiota effects is usually mucosal ecology

This mucosal ecosystem is not passive; its epithelial layer actively participates in immune surveillance. The evidence increasingly shows that the microbiota-related effects of seaweed polysaccharides ultimately converge on the mucus layer, epithelial energy metabolism, and the immune microenvironment. Studies on ulvan, fucoidan, and alginate continue to connect colonic barrier integrity, metabolic homeostasis, and inflammatory tone (30, 41, 56, 61). If seaweed sulfated polysaccharides are viewed as microbiota-friendly substrates, their main value lies not in changing one genus, but in restoring a more stable mucosal ecosystem through microbiota and metabolite remodeling.

**Figure 2:**
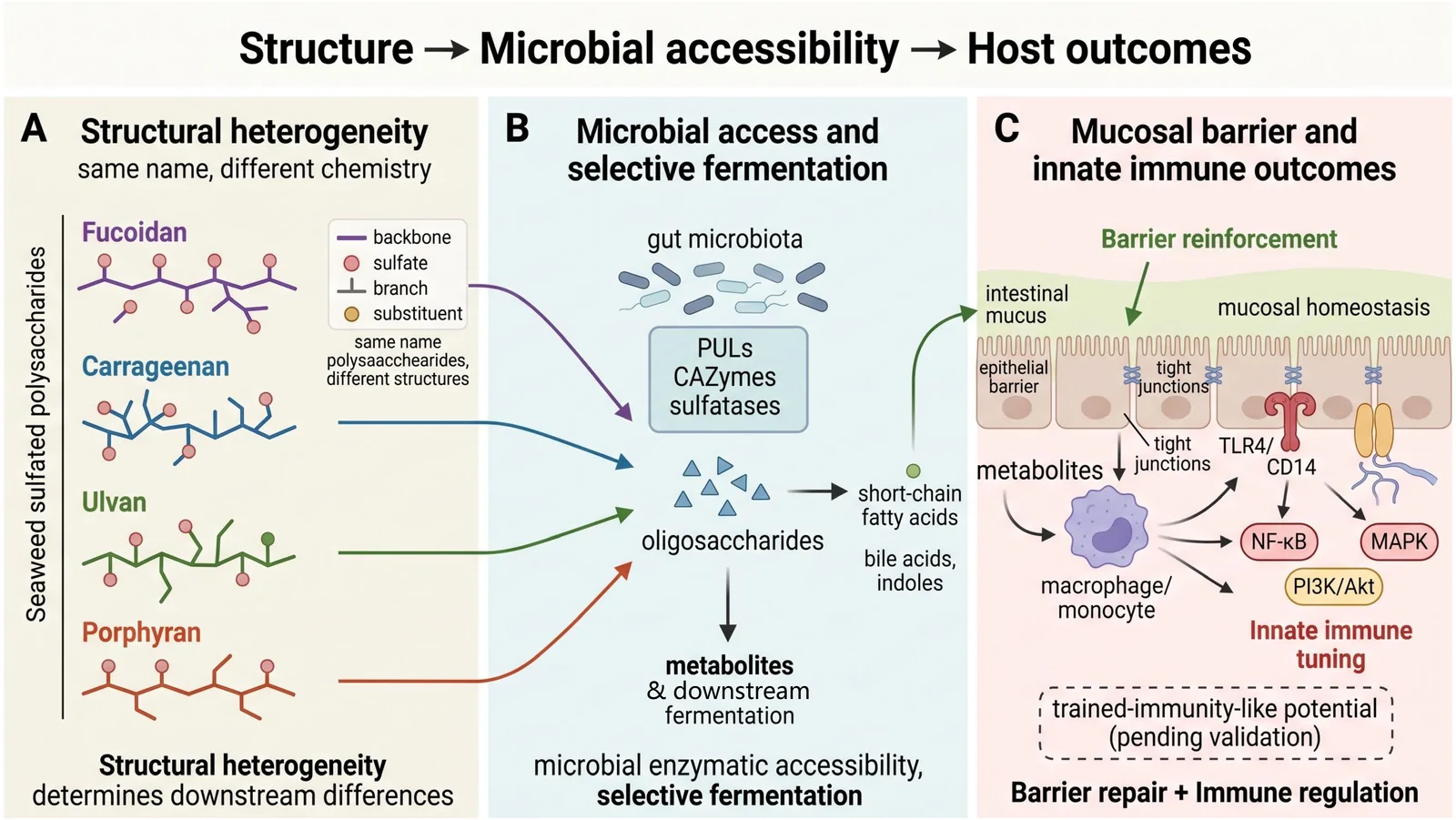
Infographic illustrating how structural heterogeneity in seaweed sulfated polysaccharides influences microbial accessibility and host outcomes across three panels: **(A)** Chemical structures of fucoidan, carrageenan, ulvan, and porphyran. **(B)** Microbial accessibility, fermentation processes, and metabolite production. **(C)** Effects on the mucosal barrier, metabolite–host interactions, and immune responses, including barrier reinforcement and innate immune modulation.

From the barrier perspective, epithelial cells are themselves innate immune sentinels. Intestinal epithelial cells express TLRs, NLRs, and NOD-like receptors, and upon sensing stimuli they can induce CCL20, CXCL8, RegIIIgamma, and beta-defensins while also influencing dendritic-cell tolerance and sIgA homeostasis through MUC2 glycosylation and antigen-sampling behavior. Once the barrier is repaired, the local antigen burden falls, thereby reducing the pressure on dendritic-cell sampling and the likelihood of excessive innate immune activation. Thus, seaweed sulfated polysaccharides primarily function to reshape the immune microenvironment, rather than merely ameliorating a single histological endpoint. (**Figure 2**).

These microbial metabolites matter because they do not merely alter community composition; they are translated into host signals through receptor-ligand pairs. SCFAs act through GPR41/FFAR3, GPR43/FFAR2, and GPR109A to influence inflammatory thresholds in epithelial cells, macrophages, and dendritic cells. Secondary bile acids signal through TGR5 and FXR to modulate macrophage and ILC function, whereas indole metabolites act as AhR ligands that support IL-22 production and barrier maintenance.

## 4 Innate immune regulatory mechanisms of seaweed sulfated polysaccharides

### 4.1 Immunostimulatory capacity of seaweed sulfated polysaccharides and training-induced immunity

In innate immunity research, RAW264.7 cells and other macrophage models remain the most widely used readout systems. Multi-omics analyses of the sulfated polysaccharide CRVP-1 from *Caulerpa racemosa* var. *peltata* suggested that CD14 may participate in TLR4-mediated NF-kappaB signaling and drive broad immunometabolic remodeling (62); the results indicate that seaweed polysaccharides do more than alter one inflammatory marker, because they can shift the overall cell state (63). Likewise, fucoidan from *Sargassum hemiphyllum* increases NO, iNOS, and COX-2 in RAW264.7 cells, confirming that at least some seaweed sulfated polysaccharides have clear immunostimulatory capacity (64).

Seaweed sulfated polysaccharides and related degradation products are not uniformly stimulatory. Depending on the molecular structure, extraction or processing conditions, and experimental model, these preparations may either attenuate inflammatory responses or promote immune activation (**Table 1**). For example, a sulfated polysaccharide from *Gracilaria lemaneiformis* showed enhanced anti-inflammatory activity after UV/H_2_O_2_ treatment, whereas carrageenan-type and xylan fractions from different red seaweeds produced distinct effects on macrophage proliferation, LPS-stimulated nitric oxide production (LPS, lipopolysaccharide), phagocytosis, and cytokine expression (65, 66). Similarly, native and partially depolymerized red-algal galactans showed different effects in RAW264.7 macrophages, with the response depending on galactan type, molecular weight, and LPS stimulation (67). Collectively, these findings show that immunological outcomes cannot be attributed to sulfation alone. Molecular weight, sulfate pattern, glycosidic linkage, and branching define the physicochemical form presented to immune cells, whereas extraction or depolymerization can modify these attributes and dose determines effective cellular exposure. Moreover, LPS-stimulated or other inflammatory models begin with pre-existing innate activation; consequently, their responses should not be generalized to unstimulated macrophages or healthy hosts. Cross-study comparisons must therefore report and account for material characterization, dosage, and inflammatory status.

**Table 1:** Overview of structural features, microbial utilization, and immune readouts of seaweed sulfated polysaccharides.

Polysaccharide	Key structural determinants	Microbial access/enzyme constraints	Representative metabolite/readout pattern	Barrier/immune functional interpretation	Evidence strength and limitations
Fucoidan	Brown-seaweed sulfated fucose-containing polysaccharides; usually alpha-L-fucose-rich but often containing uronic acids, galactose, xylose, mannose, acetyl groups, branching, and variable molecular weight.	Requires structure-matched fucoidanases, fucosidases, sulfatases, carbohydrate esterases, and PUL-like modules. Marine Planctomycetota studies illustrate substrate-specific fucoidan pathways, including GH168 endo-fucanase specificity.	Variable short-chain fatty acid and bile-acid remodeling; DSS-colitis studies link fucoidan to butyrate, bile-acid signaling, barrier repair, and reduced inflammation. Macrophage studies also show NO/iNOS/COX-2 or anti-inflammatory responses depending on source and processing.	Structure-dependent regulation of macrophage activation thresholds, gut microbiota, bile-acid signaling, mucosal barrier repair, and gut-liver/gut-brain axis-related outcomes.	Rich immune and bowel-health evidence, with strong marine enzyme evidence and emerging animal disease evidence. Gut-specific enzyme validation, batch heterogeneity, and clinical standardization remain major limitations.
Carrageenan	sRed-seaweed sulfated galactans; kappa, iota, and lambda subtypes differ in sulfate number, sulfate position, and 3,6-anhydrogalactose content. Molecular weight and degradation state are critical variables.	Carrageenases and CarPULs are subtype specific. GH16_17 carrageenases and sulfatases help explain kappa/iota/lambda selectivity, especially in recent ruminant microbiome studies.	Defined kappa/iota-carrageenan oligosaccharides can suppress LPS-induced inflammatory mediators in macrophages, whereas lambda-carrageenan may aggravate pathogen-induced colitis with reduced short-chain fatty acids, increased fecal LPS, mucus damage, and barrier disruption.	Carrageenan is not functionally uniform; native food-grade forms, degraded fractions, and defined oligosaccharides may show different inflammatory, antiviral, barrier, or model-inducing effects.	Strong structural literature and emerging animal-gut functional metagenomic evidence. Biological interpretation requires strict separation of subtype, molecular weight, degradation state, dose, and disease model.
Ulvan	Green-seaweed sulfated heteropolysaccharide, especially from Ulva species; contains rhamnose, uronic acids, xylose-containing motifs, and variable sulfate substitution. SXRG84 is a sulfated xylorhamnoglucuronan-type ulvan.	Ulvan utilization requires ulvan lyases, downstream hydrolases, and sulfatases, but gut-relevant PULs remain less resolved than porphyran or carrageenan pathways.	Constipation models show improved fecal water content, gastrointestinal transit, motilin, gastrin, 5-HT, Lactobacillus/Muribaculum enrichment, and arginine and S-adenosylmethionine-related metabolic shifts. Human SXRG84 trials suggest effects on inflammatory and metabolic markers.	Promising for motility regulation, mucus turnover, epithelial repair, local delivery, and systemic inflammatory/metabolic readouts after oral intake.	Evidence is emerging and includes early human studies, but extraction conditions, structural heterogeneity, and limited gut-enzyme validation constrain comparison across studies.
Porphyran	Red-seaweed sulfated galactan from Porphyra/Pyropia; agar-related backbone with sulfate substitutions and variable degradation state.	Porphyranases, agar-related enzymes, sulfatases, and Bacteroides-associated PULs provide a relatively clear microbe-substrate linkage; acquisition of seaweed-glycan genes by human gut bacteria has been documented.	Partially degraded porphyran increases short-chain fatty acids and enriches Bacteroides, Muribaculum, and Lactobacillus, with restored mucus thickness and tight-junction expression in DSS colitis models.	Provides one of the clearest examples of microbiota-metabolite-barrier coupling among seaweed sulfated polysaccharides.	Mechanistic chain is relatively coherent in animal colitis models, but human validation, material standardization, and class-wide generalization remain limited.

The table summarizes the relative strength of evidence along the chain from structural parameters to microbial accessibility, metabolite output, and barrier/immune endpoints; receptor networks and cell-type details are discussed in Section 4.

An additional time-dependent pattern was reported in primary porcine blood monocytes and alveolar macrophages treated with seaweed sulfated polysaccharide based *Ulva* sp and *Solieria chordalis*. All preparations produced short-term pro-inflammatory effects, and two inhibited PRRSV replication. After a 7-day washout, however, the same preparations induced a more anti-inflammatory response and no longer altered PRRSV replication. Collectively, these data indicate that the immunomodulatory effects of seaweed polysaccharides are not limited to acute stimulation; rather, they persist as a functional memory that alters the cell’s response to subsequent challenges. The transition from pro-inflammatory to anti-inflammatory reactivity upon restimulation is phenotypically consistent with a trained-immunity-like reprogramming of innate immune cells (68).

### 4.2 Signaling pathways downstream of receptor recognition: NF-κB, MAPK, and PI3K/Akt

At the signaling level, TLR4/CD14 remains the most frequently documented entry point for algal sulfated polysaccharides, and NF-kappaB, MAPK, and PI3K/Akt are the most common downstream pathways. The multi-omics study of CRVP-1 and the work on the Ulva prolifera oligomer F3 both showed that seaweed polysaccharides can regulate NO, PGE2, TNF-alpha, IL-1beta, and IL-6 via MAPK and NF-kappaB, and can also induce iNOS and COX-2 expression, indicating that they influence not only inflammatory mediators but also phagocytosis, migration, and immunometabolic state (28, 62). Other fucoidan and carrageenan fractions, by contrast, display inhibitory effects in inflammatory or oxidative-stress models, suggesting that these pathways often function as threshold regulators rather than as fixed one-way switches.

TLR4/CD14 is therefore the best-characterized, but not the only, entry route. For laminarin-like beta-glucans, the Dectin-1/CLR axis is biologically appealing; for fucoidan and carrageenan, complement, mannose receptor/DC-SIGN, and the NLRP3/NLRP6 inflammasome all deserve closer testing in mucosal models. Direct evidence for dendritic cells and ILCs is also substantially weaker than for macrophages. Structural differences may further affect endocytosis and intracellular trafficking, including receptor clustering, phagocytosis, and phagolysosomal maturation promoted by large, highly sulfated materials, whereas smaller degradation fragments are more likely to diffuse into endosomal TLR pathways but may lack sufficient crosslinking capacity.

Important gaps remain for NLRP3 inflammasome activation, caspase-1 cleavage, GSDMD-mediated pyroptosis, and the coupling between autophagy and inflammasome signaling. These processes are still largely speculative in the seaweed polysaccharide field and therefore represent some of the most valuable directions for future cell-biological work.

### 4.3 The effects of seaweed sulfated polysaccharides on oxidative stress, angiogenesis, and tissue responses

The immunological effects of seaweed polysaccharides are not limited to cytokine readouts; oxidative stress and tissue responses often change in parallel. Dörschmann et al. showed that five brown-seaweed fucoidans can modulate oxidative stress and VEGF-related processes in ocular cells, indicating that immune, redox, and vascular responses are often coupled in these materials (69). A comparable link between inflammatory signaling and cellular metabolism has been reported for defined κ/ι-carrageenan hexaoses (κ/ι-neocarrahexaoses, KCO-4). In LPS-stimulated RAW264.7 macrophages, KCO-4 reduced NO, TNF-α, IL-1β, IL-8, iNOS, and COX-2 expression, accompanied by marked changes in the cellular proteome and mitochondrial metabolic pathways (70). In a distinct tumor setting, λ-carrageenan enhanced radiation-associated ROS accumulation, caspase-3/-8 activation, and apoptosis, while suppressing cancer-cell invasion and metastasis (71). These findings show that seaweed sulfated polysaccharides exert structure-dependent modulation of inflammatory threshold, redox state, cellular metabolism, and the tissue microenvironment.

### 4.4 Regulation of intestinal disease contexts by seaweed sulfated polysaccharides

Inflammatory bowel disease provides the clearest disease-oriented evidence for seaweed sulfated polysaccharides. The available animal studies indicate that the dominant mediator may differ among polysaccharide classes, whereas restoration of the mucosal barrier represents a common downstream outcome. In a DSS-induced colitis model, partially acid-hydrolyzed porphyran from *Porphyra haitanensis* reduced disease severity, increased *Bacteroides*, *Muribaculum*, and *Lactobacillus*, elevated short-chain fatty acid production, and restored mucus thickness and tight-junction protein expression (33, 73). Fucoidan also ameliorated DSS-induced colitis and repaired the intestinal barrier, but its effects were associated with remodeling of the gut microbiota and bile-acid pool, including increased butyrate and bile acids linked to FXR and TGR5 activation; fecal microbiota transplantation further supported the contribution of microbiota-mediated mechanisms (72). Thus, porphyran and fucoidan appear to reach a similar endpoint of reduced colonic injury through distinct microbiota-derived metabolic routes.

Endotoxin- and pathogen-driven models further extend this disease context beyond chemically induced DSS injury. In a lipopolysaccharide (LPS)-challenged mouse model, an *Ulva prolifera* polysaccharide-zinc complex derived from a sulfated green-algal polysaccharide alleviated colon shortening, MPO and DAO elevation, epithelial and goblet-cell damage, and activation of the TLR4/NF-kappaB pathway (74). In a *Salmonella Typhimurium* infection model, *Undaria pinnatifida* fucoidan reduced mortality, pathogen burden, mucus-layer disruption, tight-junction loss, oxidative stress, and NF-kappaB activation, while reshaping the microbiota and metabolite profile, including increases in *Lactobacillus*, *Akkermansia*, short-chain fatty acids, and secondary bile acids (75). By contrast, dietary λ-carrageenan aggravated *Citrobacter rodentium*-induced infectious colitis, accompanied by mucus-layer degradation, increased fecal LPS, reduced short-chain fatty acids, and microbiota-dependent barrier disruption (76). These findings show that microbial or endotoxin-driven intestinal inflammation provides an important complementary model system for evaluating seaweed sulfated polysaccharides. These findings highlight microbial or endotoxin-driven intestinal inflammation as a physiologically relevant model system for evaluating seaweed sulfated polysaccharides.

Constipation represents a related but functionally distinct intestinal disorder. *Ulva lactuca* polysaccharides increased fecal water content and gastrointestinal propulsion, elevated motilin, gastrin, and 5-hydroxytryptamine, reduced TNF-alpha and IL-6, and were accompanied by enrichment of *Lactobacillus* and *Muribaculum* and changes in arginine and S-adenosylmethionine metabolism (58). Overall, current evidence indicates that seaweed sulfated polysaccharides regulate intestinal disorders in a phenotype-dependent manner: barrier repair and inflammatory restraint are central in colitis, whereas water retention, neuroendocrine motility signaling, and microbial metabolism are more prominent in constipation.

### 4.5 Human evidence and systemic regulation by seaweed sulfated polysaccharides

Human evidence is currently available mainly for SXRG84, a sulfated xylorhamnoglucuronan (ulvan) derived from *Ulva* sp. 84. In two randomized trials involving overweight or obese participants, SXRG84 supplementation was associated with reductions in selected lipid and inflammatory markers and, in one study, shifts in gut microbial composition; however, the lipid outcome was not reproduced in the crossover study, whereas lower inflammatory cytokine concentrations were observed after SXRG84 treatment (61). In a separate randomized crossover trial involving participants with inflammatory skin conditions, six weeks of SXRG84 supplementation was associated with lower circulating inflammatory cytokines, and a subgroup of participants reported improvement in skin-related symptoms (77). These studies provide early human evidence that orally administered ulvan can influence systemic inflammatory and metabolic readouts.

Increasing evidence indicates that the systemic actions of seaweed sulfated polysaccharides are mediated through gut–organ axes. In diabetic rats, *Laminaria japonica* fucoidan reduced serum triglycerides, cholesterol, low-density lipoprotein cholesterol, and hepatic lipid deposition, while reshaping the gut microbiota, increasing selected secondary bile acids, and promoting FXR and TGR5 expression, linking microbial and bile-acid remodeling to hepatic lipid metabolism (78). A gut-brain connection has also been reported in a DSS-induced colitis model with depressive-like behavior: *Laminaria japonica* fucoidan reduced colitis severity, restored colonic tight-junction proteins, altered the gut microbiota, decreased corticosterone and neuroinflammatory cytokines, and attenuated microglial activation (79). Together, these findings suggest that the systemic effects of seaweed sulfated polysaccharides are not independent disease-specific observations, but may arise from intestinal regulation that is propagated through bile-acid signaling to the liver or through microbial, endocrine, and inflammatory signaling to the brain.

Mechanistically, gut-organ axes explain how seaweed sulfated polysaccharides can produce systemic effects despite acting primarily within the intestinal lumen. Because many high-molecular-weight sulfated polysaccharides are poorly absorbed in intact form, their extra-intestinal actions are more plausibly transmitted through microbial metabolites, epithelial barrier integrity, and immune-endocrine signaling. Along the gut-liver axis, microbial fermentation can increase SCFAs, while polysaccharide-driven changes in microbial bile-acid transformation reshape the intestinal and hepatic bile-acid pool. These signals can engage FXR/TGR5 pathways, influence hepatic lipid metabolism, and reduce inflammatory tone. At the same time, improved mucus and tight-junction integrity may limit LPS translocation through the portal circulation, thereby reducing hepatic TLR4/NF-kappaB-related inflammation (80, 81). Along the gut-brain axis, SCFAs and tryptophan-derived indoles can regulate enteroendocrine activity, 5-hydroxytryptamine (5-HT)-related signaling, vagal inputs, and systemic cytokine production. These pathways provide a mechanistic basis for linking polysaccharide-induced changes in fermentation, motility, appetite regulation, neuroinflammation, and healthy aging (82, 83). Therefore, the gut microbiota serves as a signaling hub through which seaweed sulfated polysaccharides connect intestinal homeostasis with hepatic metabolism and neuroimmune regulation.

## 5 Controversies, limitations, and translational pathways

### 5.1 Reproducibility and comparability as the central challenges

Seaweed sulfated polysaccharide research has produced many positive findings, but the main barrier to comparison across papers is often not the direction of the effect, but whether the materials themselves are equivalent. Source species, season, processing, and purification route all change the composition and comparability of the samples (1–3, 5). For both reviews and primary studies, this means that origin, harvest time, extraction conditions, and fractionation route should be treated as biological variables rather than as minor methodological details.

The same problem is visible in fractionation and depolymerase studies, which show that a nominally identical material can contain substantial internal heterogeneity (2, 20, 84). If such differences are not specified, mechanistic discussions quickly lose comparability. The value of a review is therefore not simply to collect positive findings, but to convert complexity into a set of comparable structural parameters.

At the cell-biological level, this heterogeneity often appears as a trade-off among receptor binding, spatial valency, and bioaccessibility. The higher the sulfation density and branching degree, the more likely the molecule is to promote multivalent binding and receptor clustering at the cell surface and thereby raise the signaling threshold; however, the larger the molecular weight, the harder it is to cross the mucus layer, and the more the effect depends on local retention or microbial pre-processing. By contrast, low-molecular-weight oligosaccharides diffuse and are taken up more easily, but may not preserve enough multivalency. Structural parameters therefore do not simply decide whether activity exists; they jointly shape the final phenotype by balancing affinity, valency, and transport route.

### 5.2 Extraction and degradation are themselves functional variables

For seaweed polysaccharides, extraction is not a neutral operation but the starting point of structural remodeling. Acid, alkali, enzyme, ultrasound, microwave, and high-pressure routes can change molecular weight, sulfate distribution, and impurity profiles, and they can also alter subsequent microbial utilization and cellular responses (3, 34–39). In some cases, moderate degradation is actually more favorable for colonic utilization and tissue responses, as shown by partially acid-hydrolyzed porphyran and enzyme-treated sulfated galactan preparations that display clearer microbial selectivity (33, 55).

Future studies should not mix untreated raw materials with degraded oligosaccharides. The former are more likely to reflect the gel-forming, adhesive, and barrier properties of the material itself, whereas the latter more clearly capture receptor recognition and microbial enzymology. Because the two material classes serve different purposes, they should be tested in different models and judged by different endpoints.

### 5.3 Safety and bioaccessibility are two unavoidable translational thresholds

Seaweed polysaccharides are often assumed to be natural, safe, and edible, but realistic translational assessment cannot rely on intuition alone. Salt background, sulfation degree, long-term high-dose exposure, and processing route all need systematic evaluation before a material is advanced (34, 39, 40). Carrageenan and some highly sulfated samples, in particular, do not have identical risk boundaries in food, pharmaceutical, and materials contexts, so they should not be bundled under a single “natural product” label.

Many seaweed sulfated polysaccharides also do not rely on systemic absorption; rather, they act locally in the colon through the microbiota and epithelial cells. This is advantageous for local applications because local action can reduce systemic exposure, but only if product design matches the site of action. If the target is the colon, the formulation should favor digestibility, fermentability, and local retention; if the target is the upper digestive tract or systemic inflammation, a clearer delivery strategy is required.

From a cell-biological perspective, the first real translational obstacle is bioavailability and local accessibility. The limited mucus penetration of high-molecular-weight, strongly sulfated polysaccharides can be explained by size exclusion, electrostatic effects, and hydration-shell formation. Intestinal mucus is a hydrated mucin network whose pore structure restricts the diffusion of large polymers; strong sulfation increases negative charge density and enlarges the effective hydrodynamic radius of the molecule, which can promote retention within the outer mucus layer rather than free diffusion toward epithelial receptors (85–87). Negatively charged mucins containing sialylated and sulfated glycans may further create charge-dependent repulsion or steric trapping for similarly anionic macromolecules. Partial microbial or enzymatic depolymerization reduces molecular size and may expose receptor-accessible oligosaccharide motifs, making indirect interaction through microbial cleavage products more plausible than direct epithelial penetration by intact polymers. Mucus-penetration assays, fluorescent labeling, ex vivo explants, and epithelial-immune-microbiota coculture systems are therefore needed to distinguish the contributions of intact polymers, degradation fragments, and microbial metabolites.

### 5.4 Framing translation around functional scenarios rather than single molecules

Current translational studies on seaweed polysaccharides are no longer limited to bioactivity itself; they increasingly point toward hydrogels, microcapsules, edible films, and local delivery systems. The work on ulvan hydrogels, fucoidan-based hydrogels, alginate encapsulation, and carrageenan films shows that these molecules simultaneously possess material properties and functional properties (38, 56, 88–90). The real question is not whether they can be turned into materials, but which form should be used, in which anatomical site, and for which disease context.

A more realistic translational roadmap can therefore be divided into three paths: first, as gut-health and prebiotic functional ingredients for IBD, constipation, and barrier maintenance; second, as delivery platforms or composite carriers for local release and tissue adhesion; and third, as structural ingredients in functional foods or nutraceuticals for chronic-disease management and healthy aging. All three routes require structural standardization, dose definition, and human evidence. In addition, the translational pathway would benefit from more structured information and advisory frameworks to align material characterization, extraction choices, and application scenarios, analogous to how decision-support systems facilitate adoption in other agricultural settings (91, 92).

## 6 Conclusions

The value of seaweed sulfated polysaccharide research lies in its position at the intersection of structure, the microbiota, and mucosal immunity. Fucoidan, carrageenan, ulvan, and porphyran, together with related alginate and laminarin, represent different backbone and sulfation patterns and therefore different modes of microbial utilization and tissue response. Based on the current evidence, the most mature scenario remains intestinal mucosal immunity, especially IBD, constipation, and barrier injury, whereas innate immunity and systemic inflammation are more often characterized by structure-dependent modulation.

Future work can productively focus on five prioritized directions. In the short term, structural standardization should be moved upstream, with source species, season, extraction process, fractionation method, molecular weight, sulfation parameters, and endotoxin/impurity status treated as mandatory reporting items. Second, mechanistic studies should move away from single-point markers toward the continuous chain linking microbiota, metabolites, mucus layer, epithelial cells, and immune cells, supported by multi-omics, histology, and enterotype-aware designs. Third, human studies should be strengthened, especially in IBD-like barrier dysfunction, functional constipation, inflammatory skin disease, and metabolic disease, while remaining cautious about clinical efficacy claims. Fourth, translational research should prioritize achievable applications such as standardized gut-health ingredients and local barrier-support formulations before advancing longer-term goals such as precision enterotype-based applications or systemic inter-organ-axis targeting. Fifth, trained-immunity assessment should be treated as a dedicated experimental framework rather than inferred from transient cytokine changes.

For candidate materials such as seaweed polysaccharides, future trained-immunity studies should place restimulation, adequate washout, epigenetic changes, metabolic reprogramming, and *in vivo* transferability within the same pre-registered design so that transient inflammatory fluctuations are not mistaken for trained immunity.

Seaweed sulfated polysaccharides are already equipped with clear immunological interfaces, but they still require stricter structural definition and clinical validation. The next key step is to define which disease scenarios, mechanistic routes, and product forms are most appropriate for each polysaccharide class.
